# Ontology-driven integration of advertised and operational capabilities in robots

**DOI:** 10.1038/s41598-025-16649-3

**Published:** 2025-10-02

**Authors:** Muhammad Raza Naqvi, Arkopaul Sarkar, Farhad Ameri, Linda Elmhadhbi, Thierry Louge, Mohamed Hedi Karray

**Affiliations:** 1 Laboratoire Génie de Production (LGP), Université de Technologie Tarbes Occitanie Pyrénées (UTTOP), 47 Av. d’Azereix, 65000 Tarbes, France; 2 Department of Physics, Georgetown University, 37th St NW, Washington, DC 20057 USA; 3 School Of Manufacturing Systems and Networks, Ira A. Fulton Schools of Engineering, Arizona State University, 6075 Innovation Way W, Tempe, AZ 85283 USA; 4 INSA Lyon, Université Lumière Lyon 2, Université Claude Bernard Lyon 1, Université Jean Monnet Saint-Etienne, DISP UR4570, 69621 Villeurbanne, France

**Keywords:** Ontology, Capabilities, Robotics, Knowledge technologies, Manufacturing, Computer science, Information technology, Software

## Abstract

The adaptability of robotic systems is expanding the horizons of manufacturing flexibility. However, fully leveraging the potential of these systems poses considerable challenges. A key requirement is the ability to understand and model their diverse capabilities through a standardized and semantically well-defined framework. In this paper, we introduce the Robotic Capability Ontology (RCO), developed through a systematic investigation of various types of robotic capabilities, including those related to function, quality, and process performance. We define two types of capabilities: Advertised capabilities, as specified by manufacturers, and Operational capabilities, which reflect real-world performance. The RCO framework provides an ontology-based approach to representing these capabilities in a structured and interpretable manner. Within the manufacturing context, RCO serves as a reference ontology that bridges manufacturer specifications and empirical performance data to support more accurate, explainable, and interoperable representations of robotic capabilities.

## Introduction

We are on the leading edge of a robotics revolution that promises to transform the world in multiple dimensions as the new era of modern technology takes shape. Robotics has wholly transformed our society and economies^1^ by redefining healthcare^2^, manufacturing^3^, education^4^. Other emerging industries^5^, and by introducing competence, dependability, and even innovation on an impossible scale. Nowadays, robots can accomplish pre-defined tasks and evolve and grow, providing a range of capabilities that extend past their initial design and enabling them to meet new challenges and perform complex operations in dynamic environments.

As robotic capabilities change from static to dynamic, it is essential to fully understand and accurately formalize these capabilities when they are set up and used. Moreover, it is essential to highlight the difference between robots’ function, quality, and process characteristics. This knowledge is essential for improving robot design, implementation, and use across industries. To define robot capabilities, it is necessary to use the advertised specifications or manuals provided by the robot’s manufacturer to accurately determine the robot’s different specifications regarding its reach, repeatability, precision, footprint, etc. An ontological framework can help formalize, identify, and distinguish these capabilities.

In this paper, we demonstrate the design of an ontology that precisely captures these notions of the robot from its design specifications. We propose creating an ontology that accurately captures these robot notions based on its specifications. Formalizing these concepts in an ontology entails developing a structured framework that not only categorizes the various aspects but also demonstrates their interdependence and impact on robotic systems. By doing so, we can gain a more comprehensive understanding of robotic capabilities, including both innate quality functions and process characteristics.

In this paper, we concentrate on three specific research questions concerning robotics:

**(Q1)** How do robot manufacturers’ advertised capabilities vary when compared to their operational capabilities?

**(Q2)** Why is it important to differentiate between capabilities and capacity when talking about robots, and precisely how does that difference affect the development and implementation of robots?

**(Q3)** What are the most important factors to remember when creating an ontological framework that accurately captures and represents robot capabilities?

The remainder of this paper is structured as follows:

A concise summary of the scientific studies conducted by robotics researchers related to capabilities is presented in “Related work”. This section also discusses how capability modeling has a significantly more significant influence on the future development of robotics. Additionally, it examines the significance of ontology and how it can assist in modeling the capabilities of robotic systems; we perform a comparative Analysis of Robotic Ontologies with RCO to showcase the importance and need to formalize advertised and operational capabilities. The importance of formalizing robotic specification (advertised and operational) capabilities is emphasized in “Advertised and operational capabilities”. “Understanding the complexity of conceptualizing advertised and operational capabilities” discusses the formalizing of robotic capabilities in the context of the manufacturing industry 4.0 paradigm. “Robotic Capability Ontology (RCO)” explains the concept of ontology and ontology development method for formalizing robotic specifications using MSDL and how we model robotic capabilities. “Ontology validation using SPARQL”, we validate the ontology model using SPARQL Queries, and “Application of RCO” prescribes the relevant applications of RCO. Finally, “Conclusion” is about conclusion and future works.

## Related work

The remarkable advancements in robotics are not limited to automating tasks; they also involve developing robots with abilities that closely resemble and sometimes even surpass human capabilities.^6–10^.The rise of more powerful robotics, integrated with cutting-edge AI, has blurred traditional distinctions^11–17^. The diverse characteristics of robotic capabilities complicate the decision-making process and vary depending on the environment, scenarios, and tasks. As noted, “technology and society have changed what robots can do” (https://www.weforum.org/agenda/2023/07/robots-ai-help-humans-at-work/).

Enhanced software and controllers have allowed robots to adapt their functions based on specific circumstances, while globalized mass production tailored to market needs has driven standardization and modular design for task-specific customization. These shifts influence what robots can achieve both now and in the future. These advancements in robotic capabilities are highly promising but bring challenges related to real-world applicability and adaptability. While enhanced AI integration enables robots to perform complex tasks, it also creates a gap between the capabilities advertised by vendors and those that can be consistently achieved in operational environments.

This gap highlights the need for frameworks that explicitly differentiate between idealized vendor specifications and real-world functionality. This distinction is crucial for reliable decision-making and effective task allocation, particularly in diverse and unpredictable environments such as manufacturing. Next, we will look into various works that contribute valuable insights into adaptability and modular functionality in robotics. However, these studies stop short of addressing the critical differentiation between the capabilities advertised by vendors and the actual operational performance of robots in real-world environments.

In manufacturing, capability is critical whenever machines, tools, or equipment are involved, as it directly affects the quality and efficiency of production processes and outcomes. The capability of a machine determines its precision, accuracy, and reliability-all of which play a pivotal role in meeting production standards^18^.

It is essential to comprehend the concept of “capability” before diving into the specifics of robotic capabilities. According to ISO 15531-31^19^, capabilities and capacities are distinguished on a qualitative-quantitative axis, where capacity is a quantitative concept exemplified by product throughput, and capability is qualitative. According to MERREL et al., “a capability of an entity is, intuitively, the potential for that entity to do something useful”^20^.

The word “capability” is frequently used in daily life to refer to software capabilities, human capabilities, the capabilities we will acquire through learning (e.g yoga classes), and so on. Some entities can carry out actions that are useful or wanted. For example, my passport allows me to travel and meet people from different cultures. My football boots provide me with the comfort and support to play football.

For example, in robotics, a robot vacuum cleaner can clean the floor all by itself, making it easier to keep the house tidy. When choosing actions and attempting to achieve objectives, it is helpful to be aware of the possibility of such activities. Thus, it is critical to know what accessible actions an entity can take.

Sabbagh and Ameri defined “capability” as the inherent potential of diverse resources in generating benefits for various stakeholders^21^, while Sarkar and Sormaz argued that capability, capacity, and competency are mainly used with interconnected connotations^22^. Francesco and Stefano talk about what capabilities and capacities entail and their similarity to each other in terms of functionality and how they can be differentiate as they state, “capacities essentially indicate how the corresponding capabilities can be practically implemented.” Also, they perform different analyses using the top-level ontology Descriptive Ontology for Linguistic and Cognitive Engineering (DOLCE) as a framework^23^.

Let’s take as an example the automobile painting robots^24^, an industry that gives importance to detail, accuracy, and productivity to manufacture a quality vehicle. These robots, which are utilized in this industry, have high specifications in terms of precision, uniformity, and speed. Additionally, environmental conditions within the factory, such as temperature and humidity, affect the paint’s viscosity and drying time, which can alter the robot’s performance. Over time, these capabilities may degrade due to wear and maintenance quality. Such environmental and maintenance conditions are seldom replicated in the controlled environments used for testing.

The manufacturers who advertised this robot as their product have a particular precision capability to apply paint consistently, and having these capabilities improves the overall performance of the production line. However, using these robots on actual automotive assembly lines reveals challenges not typically encountered during controlled tests^25^. The variability among automobile models and their surfaces may influence the robot’s precision^26^.

Specifically, robots tested on flat surfaces often struggle with the curves and edges typical of various car models and materials. Manufacturers claim that robotic arms demonstrate improved accuracy in assembly tasks^27^. However, their precision in executing specific tasks may be affected by various factors, including assembly line speeds and environmental conditions^29^.

For instance, AGRI robots^30^ are widely used in agriculture for efficient crop harvesting under optimal conditions^31^. However, their performance tends to deteriorate in less favorable environments, such as uneven surfaces and varying crop densities. Healthcare assistive robots^32^, particularly those aiding surgical procedures, face significant challenges due to unforeseen complications or the complexities of human anatomy and varying surgical techniques that may not be accounted for during the manufacturer’s optimal testing^33^. Warehouse sorting robots^34^, touted for their exceptional sorting capabilities, encounter difficulties in disorganized warehouse settings that involve a wide variety of item shapes and weights^35^. Service robots^36^, while demonstrating efficacy and interactivity under controlled conditions, may struggle with the unpredictable dynamics of human interactions and the diverse inquiries presented by consumers in real-world service environments^37^.

These examples illustrate a significant gap between advertised and operational capabilities typically tested in controlled settings; the advertised specifications often overlook the complexities and unpredictability inherent in practical applications. This disparity underscores the importance of understanding robotic capabilities in actual operational environments, particularly in sectors like manufacturing that present numerous challenges and operational variables. Foo et al. focus on the robotic disassembly of Waste Electrical and Electronic Equipment (WEEE), utilizing machine learning frameworks to enhance part identification accuracy based on past disassembly experiences^38^. Pradeepani et al. enhance robot-human interaction by integrating commonsense knowledge, employing the BERT model to improve task responsiveness and adaptability in specific domains, such as kitchen environments^39^. Frank et al. present a prototype-based skill model for robots that emphasizes reusability and extensibility via a domain-specific language, enabling users to compose complex robotic skills from modular components^40^. Oriol et al. propose an ontology-based manipulation framework for autonomous service robots, which enhances adaptability through situational awareness across perception, planning, and execution stages^41^. Elin et al. introduce an ontology-based approach for programming dual-arm robotic systems, emphasizing synchronized motion and skill transfer across different robot kinematics, user-friendly interfaces, and program transformations^42^.

Several frameworks, such as CAPILANO^43^, PROSA^44^, MANDATE^45^, MASON^46^, RAMI 4.0^47^, and MARCO^50^, provide valuable insights into aspects of robotic capabilities and resource allocation, including disassembly efficiency, adaptability through ontology-based frameworks, and synchronized dual-arm programming.

However, they do not specifically address the problem of advertised capabilities by the vendor and how they vary in the operational environment. Also, to the best of our knowledge, no ontology in robotics considers formalizing these notions. Table 1 compares various robotic ontologies across different domains, including manufacturing, with a particular emphasis on task allocation based on robotic capabilities.

This contrasts with other ontologies, such as IEEE Standard Ontology for Robotics and Automation (ORA), Core Ontology for Robotics and Automation (CORA), Extension of CORA (CORAX), Robotic Service Ontology Specification (RoSO), OWL Urban Search and Rescue, and Sensor Ontology, while valuable in their contexts related to robotics, do not address the critical differentiation between advertised and operational capabilities.

**Table 1:** Comparative analysis of existing robotic ontologies.

Ontology name	Scope	Application	Use case
CAPILANO^43^	Task allocation	Manufacturing	Capability-based task allocation for heterogeneous resources line-less mobile assembly system
PROSA^44^	Semantic representations	Smart manufacturing	Semantic representations intended for smart manufacturing
MANDATE^45^	Product, process and resource paradigm	Manufacturing	International standard for representing manufacturing data
RAMI 4.0^46^	Reference architecture of technical assets	Manufacturing	Manufacturing life cycle
MASON^47^	Entities and operational resources	Manufacturing	Ontology for manufacturing domain
BaSys 4.0^48^	Combined basic and slave capabilities	Manufacturing	Task allocation to individual resources
C41^49^	Resource capabilities matching	Resource functionalities	Resource capability matching
MaRCO^50^	Combined capabilities from combination of resources	Resource allocation	Process and resource combined capabilities
RoSO	Services via commons ontology library	Service environments	Builds robotic services foundation
IR DisAssembly Capability OWL^51^	DisAssembly Capability	ReManufacturing	Robotic DisAssembly
OWL Urban Search and Rescue^52^	Urban rescue robots	Rescue missions	Focuses on victim identification, situational awareness
Open Robot Ontology (ORO)^53^	General robotic systems, ROS-compatible	Home interactions	Enhances human-robot interaction
KnowRob^54^^55^	Reasoning from sensor data	Home robotic tasks	Supports advanced machine learning for interaction
RoboDB^56^	Robot component database	Robot design	Organises physical characteristics for design
OASys^57^	Autonomous system engineering	Robot/process control	Unifies conceptual and engineering aspects
IEEE ORA^58^	Robotics and automation	Robot standardisation	Standardises robotics knowledge
CORA^59^	Robotic system complexity	Broad robot definitions	Represents robots by functionality
CORAX^60^	Extends SUMO and CORA	Industrial settings	Focuses on design, environment interactions
Robo Design^61^	Hardware design for inspection robots	Mobile systems for building inspection	Integrates building features with design
SOHO^62^	Modeling human–robot collaborations	Interactive environments	Comprehensive modeling of HRC scenarios
RPARTS^60^	Robot parts specification	Component modeling	Details robot parts for design
POS^60^	Robot poses	Spatial modeling	Improves spatial accuracy
ADROn^62^	Automatic robot design	Automatic design	Links actions to structural parts
Robot Ontology^63^	Mobile robot systems	Locomotion focus	Details locomotion subsystems
SOMA^55^	Decision making in contexts	Various platforms	Integrates contexts, supports learning
NEPs Ontology^64^	Ethical behavior modeling	Ethical alignments	Structures ethical decision-making
OCRA^65^	Robot planning	Supports reasoning about collaborations and plan	Collaborative robot actions and plans

Therefore, RCO is proposed as a reference ontology that formalizes the robot’s advertised and operational capabilities, specifically within manufacturing settings.

Our focus remains on industrial manufacturing environments, emphasizing semantic precision in capturing the robot’s advertised and operational capabilities.

## Advertised and operational capabilities

We contend that advertised capabilities relate to inherent functions or capabilities integrated into an entity, product, or process throughout its conceptualization and engineering phases. Due to varying contexts, these capabilities may deviate from the system’s actual operational capabilities. These functionalities are frequently developed to predict users’ needs, leading to a smoother, more intuitive, and more efficient user experience. The origins of advertised capabilities can be attributed to historical product design concepts that prioritized comprehending and anticipating consumer needs^66–70^. Regarding the advertised capabilities, we state that there is a clear distinction between an object’s intended use and the actual operation ways.

There is a clear difference between the advertised capabilities specifications calculated by the manufacturer who created the product and those realized during operational use. Manufacturer tests are performed in a very optimal and controlled environment, which means the manufacturing testing facility is substantially different from where the robot will be put in the operational environment. Therefore, there is a need to draw a line between advertised and operational capabilities.

**Fig. 1:**
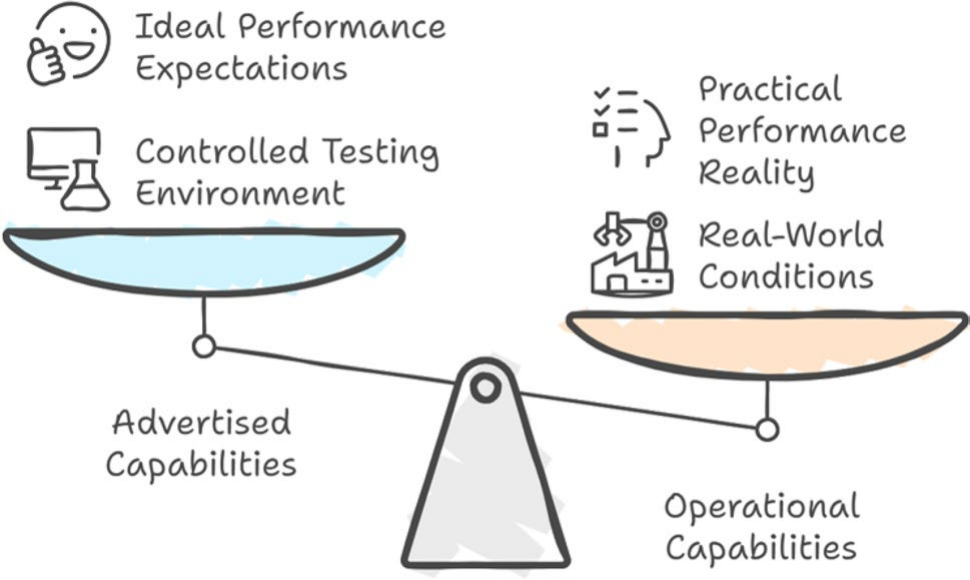
Robotic capabilities measurement process in two different contexts.

Figure 1 shows how both capabilities are measured in vendor testing facilities and end-user operational cases using the same robotic arm. Consequently, there can be a significant gap between the capabilities stated in official specifications and the robot’s operational performance. This discrepancy highlights the need for more comprehensive testing methods that better reflect the complexities of real-world industrial settings. It raises significant concerns about the reliability of the manufacturer’s claims and the need for rigorous testing methods that better capture the intricacies of real-world industrial environments, such as the Aerospace Industry. Research into robot-based manufacturing for automated aircraft assembly (AAA) began roughly three decades ago.

One significant project was the Automatic Wing Box Assembly (AWBA) demonstration cell developed by Airbus UK^71^. This system features two distinct robot setups capable of precisely positioning 6-m-high ribs into spars with an accuracy of +/- 0.5 mm and drilling and fastening skins to ribs. This evaluation, conducted in an operational environment at the Airbus UK facility, revealed positioning inaccuracies in the serial link articulated robot, causing drilled holes to fall outside Airbus’s acceptable specifications. Further testing at AT Nutech in France at a vendor testing facility also demonstrated similar inaccuracies^72,73^.

Following Airbus UK’s, another test showed that even with calibrated kinematic models, the robots could not achieve the ± 0.2 mm absolute accuracy, and their performance was adversely affected by temperature changes.

This example underscores the critical need to distinguish between the actual performance of robotic systems and the capabilities advertised by vendors, especially in high-stakes industries like aerospace. While tolerances might be acceptable on paper, the real-world implications of minor deviations in repeatability and precision can be significant. In the cases of the Airbus UK and AT Nutech tests, the discrepancies in robotic positioning, particularly in precision and repeatability, led to outcomes that failed to meet strict aerospace specifications.

Such findings highlight the importance of rigorous, real-world testing environments to honestly assess the capabilities of robotic systems, ensuring they meet the necessary standards for quality and reliability in critical applications.

Moreover, during the COVID-19 pandemic in February 2020, the Hong Kong Innovation and Technology Bureau allocated HKD 800 million under the Anti-epidemic Fund to support the research (https://www.info.gov.hk/gia/general/202005/20/P2020052000356.htm), development, and production of reusable masks for public use. This effort involved Hong Kong-based textile companies, including Crystal International Group, which operated a production line in Vietnam, and TAL Apparel, with smaller production lines in Hong Kong. These companies repurposed their existing production lines, designed initially for sewing and cutting undergarments, to produce masks.

Manufacturers assumed that machines with good performance in sewing and cutting undergarments and could maintain the same precision when creating masks. However, this assumption proved incorrect. The technical requirements for sewing and cutting masks differed significantly, and the machines struggled to adapt to the new specifications. As a result, the masks produced were poorly shaped, resembling undergarments rather than suitable face coverings, and people refused to wear them, led to a huge loss of finances (https://hongkongfp.com/2020/06/02/cumask-hongkongers-all-got-free-masks-heres-why-is-nobody-wearing-them/).

This case illustrates the challenges of adapting specialized manufacturing equipment to meet different product requirements in response to unexpected crises.

## Understanding the complexity of conceptualizing advertised and operational capabilities

Creating a physical product after going from the required specifications to the design is difficult. Apart from being technically challenging, this difficulty is also intellectual since it requires translating conceptual ideas into practical implementations.

Acknowledging this complexity is necessary for correctly reflecting the complex aspects of product development in any framework. The testing step is crucial in the manufacturing industry. It verifies the design of a certain manufactured product and provides the specifications that are advertised further as the product’s capabilities.

Although tolerance levels can be responsible for some differences between the advertised capabilities and those actually realized in the processes in the operational environment, there are situations in which the deviation is greater than these tolerances. This may result from several factors, such as the conditions of the environment, the user’s actions, or the gradual accumulation of wear and tear over time.

We describe why modeling robotic capabilities is critical in differentiating advertised and operational capabilities, especially in industrial use cases. Here, ‘modeling’ refers to the methodological approach of capturing and analyzing these variances to represent product performance realistically. We advocate for a comprehensive approach to conceptual modeling, which involves understanding the nuances of advertised and operational capabilities and accounting for potential anomalies beyond normal tolerances.

By employing such models and fostering transparency, stakeholders can better understand and manage expectations regarding product capabilities. This will benefit users with more accurate performance assessments and aid manufacturers in refining product designs:

- Automated decision-making towards trustful and flexible manufacturing, promoting reconfiguration in production lines^74^.
- Modeling these capabilities can guide the selection of appropriate robots for specific tasks to improve productivity^75^.
- Robots are costly, and a lack of knowledge or modeling of their capabilities can lead to inefficient usage and diminished resources^76^. For example, if a robot’s reach or precision is incorrectly represented, it may be assigned tasks that it cannot accomplish adequately, resulting in inefficiency and waste.
- Robots can endanger human beings if employed outside their capabilities or for jobs not built for that particular task^77^. For instance, understanding a robot’s lifting capability is crucial to prevent it from being overloaded, which could lead to equipment failure or even harm human operators and other equipment.

In the modern era of flexible production systems, robotic capability modeling has grown progressively relevant when robotics is anticipated to carry out various activities^78–80^. In this context, modeling robotic capabilities helps lead the development of adaptable robotics capable of effortlessly shifting among diverse jobs. It influences robotics’s development, installation, and secure functioning in industries, impacting productivity, trustfulness, cost efficiency, and security.

To do this, we must formalize the idea of advertised and operational capability in robotics, collecting advertised capabilities via the robot’s manufacturer design specification or robot manual and measuring operational capabilities in an operational environment. An ontology can offer the framework for recognizing and distinguishing these capabilities, enabling researchers to develop and deploy more flexible robots.

### Revealing operational limitations through comparison with advertised capabilities of the NED-2 robot

As a use case, we used the Commonsense Knowledge & Hybrid Artificial Intelligence for Trusted Flexible MAnufacturing 4.0 (https://chaikmat-anr.uttop.fr) (CHAIKMAT) production line, NED-2 Robot (https://niryo.com/products-cobots/robot-ned-2/) in the small setting to model different specifications of a robot. We extract robot specification from its manufacturer’s specification Manual (https://docs.niryo.com/product/ned2/v1.0.0/en/index.html) shown in Table 2.

**Table 2:** Advertised specifications of NED-2 robot.

Parameters	Measurement label	Measurement unit
Weight, payload	Mass	lb
Repeatability, precision, footprint	Length	Inch
Joints range	Angle	Degree, radian
Joints speed limit	Linear and rotational	Radian/s
Dimension of the standard box	Area	cm
Torque	Torque	kg cm
Pressure	Force	kPa
Diameter	Diameter	Inch

**Fig. 2:**
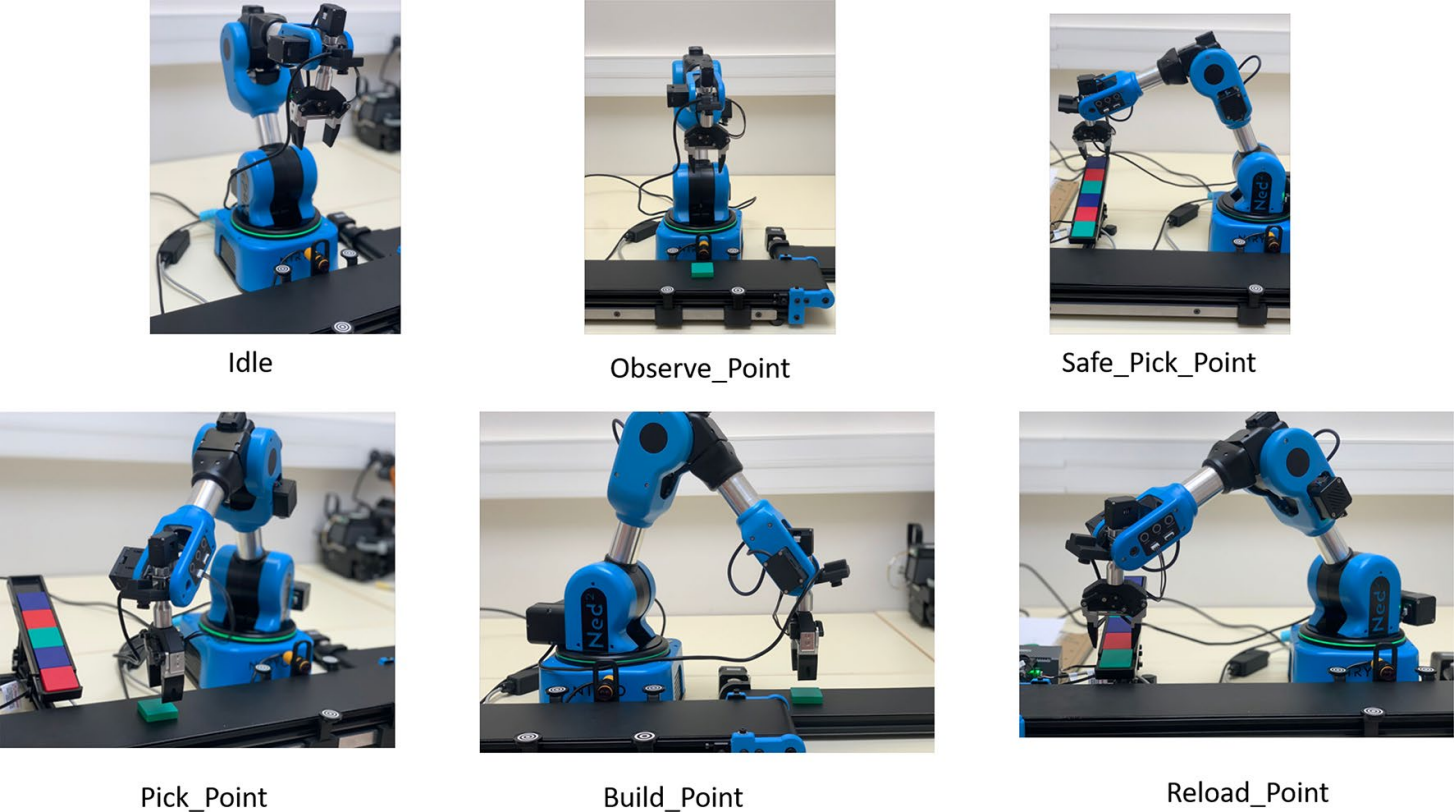
CHAIKMAT production line.

We use these parameters to model NED-2 advertised specification with repetition as repeatability capability in a use case scenario where the robot is deployed to perform handling and transportation of Lego blocks as shown in Fig. 2.

**Fig. 3:**
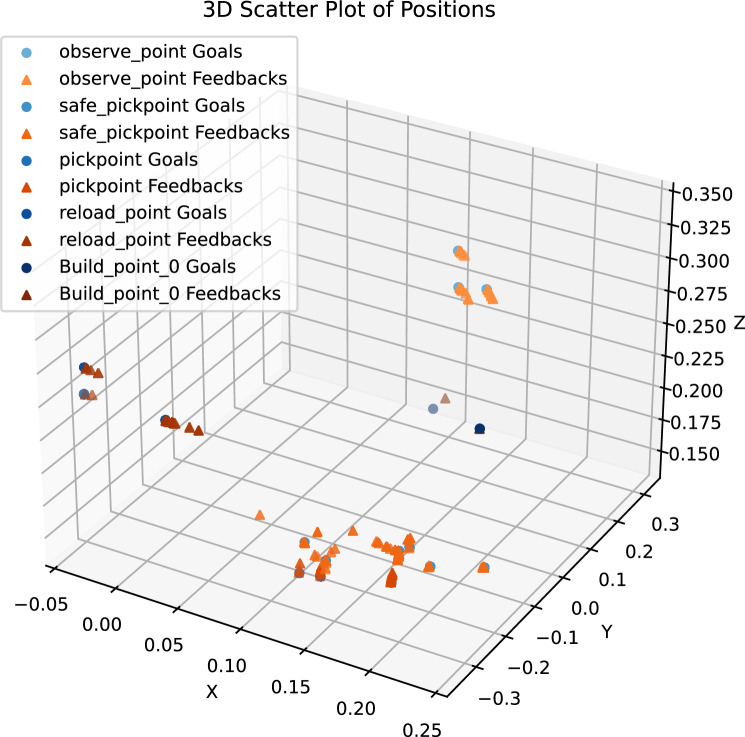
Repeatability measurement process results in 3-D space.

Ned 2 robot moves between different points; data is collected as goal and feedback; goal means the given position for the robot to go and feedback where the robot went. The repeatability is calculated as the standard deviation of feedback positions that assesses how well a robot can reach the same position under a consistent environment. The results of the repeatability measurement process at every point are shown in Fig. 3.

It helps understand how closely the feedback matches the intended goals in different operational scenarios, providing insight into the precision and effectiveness of robotic actions within these coordinates.

## Robotic capability ontology (RCO)

The RCO (https://industryportal.enit.fr/ontologies/RCO/) is a reference ontology that formalizes the capabilities of robots and considers the distinction between what manufacturers claim their robots can do and what they can achieve in practice. In the following section, we discuss RCO’s development process.

### Ontology development methodology

Various ontology development methodologies^81^ are proposed in the literature^82–85^, including OntoClean, METHONTOLOGY, and NEON, highlighting their approaches and applications in different contexts.

According to Noy and McGuinness^86^, “ there is no one correct way or methodology for developing ontologies”. Also, they state, ”there is no one correct way to model a domain- there are always viable alternatives. The best solution almost always depends on the application that you have in mind and the extensions that you anticipate”.

We utilize the 101 Ontology Development Methodology^86^ due to its recognition within the scientific community and its ability to facilitate the reuse of existing terms and concepts.

**Fig. 4:**
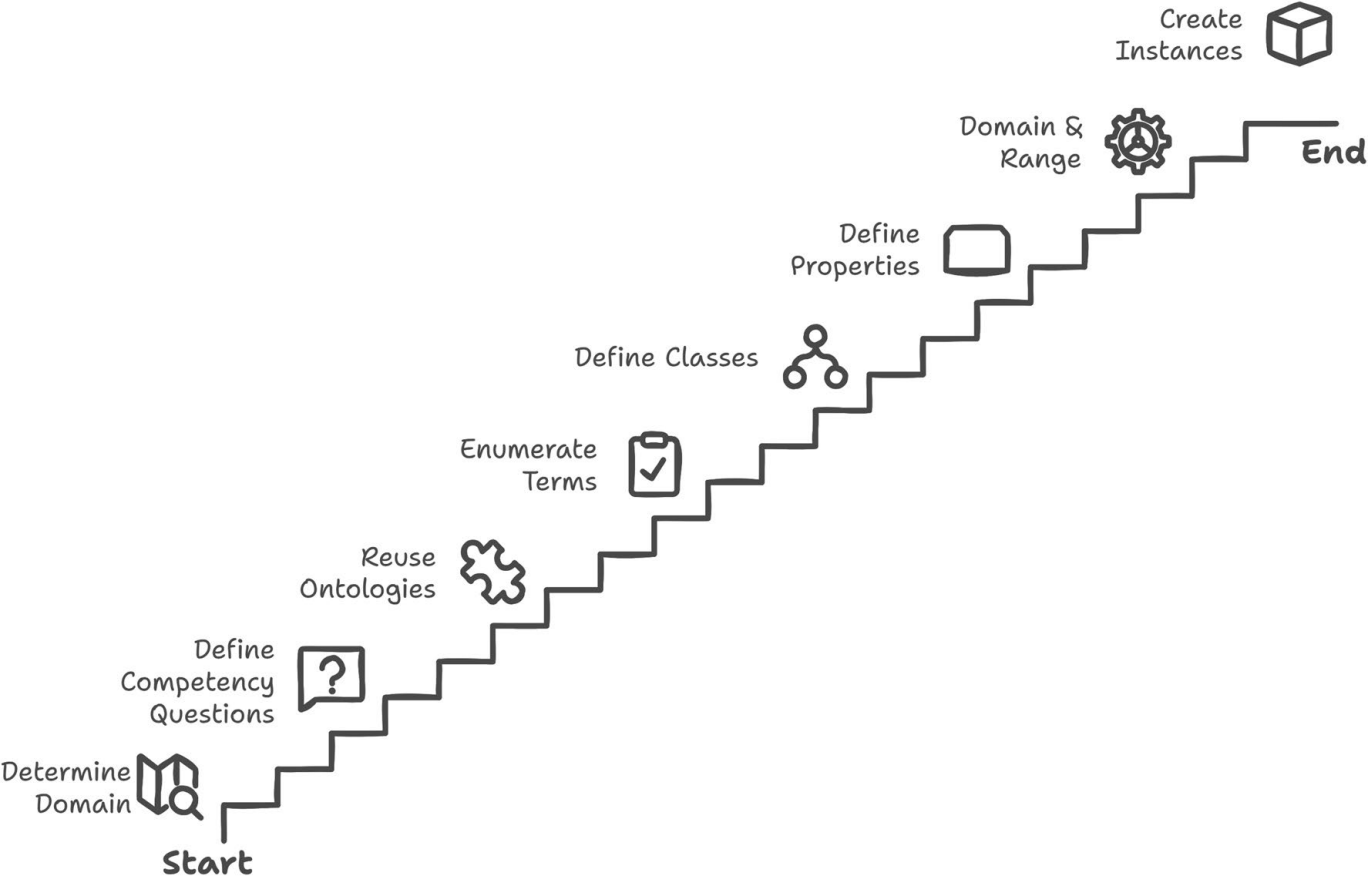
101 ontology development methodology^86^.

The 101 ontology development methodology involves the following steps as illustrated in Fig. 4, which guide the structured development of ontologies and the reuse of existing terms:

1. Determine the domain and scope of the ontology
2. Define Competency Questions (CQ,s)
3. Consider reusing existing ontologies
4. Enumerate important terms in the ontology
5. Define the classes and the class hierarchy
6. Define the domain and range of properties
7. Define the properties of classes
8. Create instances

The domain of our ontology is manufacturing, with a scope centered on distinguishing and categorizing advertised versus operational capabilities in robotic systems within a production environment.

This RCO classifies capabilities based on real-world conditions, defining relevant metrics and attributes for precise capability assessment and establishing relationships that capture the dependencies between advertised specifications and actual operational performance. By concentrating on actual, concrete examples, we illustrate how our ontology addresses practical issues that emerge when deploying robots in a production environment.

When developing our ontology, we chose to emphasize the practical value of our work by using real-world scenario-based competency questions.

Next, we define competency questions:

- CQ1: What are the key differences in how advertised and operational capabilities are categorized in robotic systems?
- CQ2: What metrics and attributes define the measurement of advertised and operational capabilities?
- CQ3: What relationships and properties are associated with advertised and operational capabilities?

**Fig. 5:**
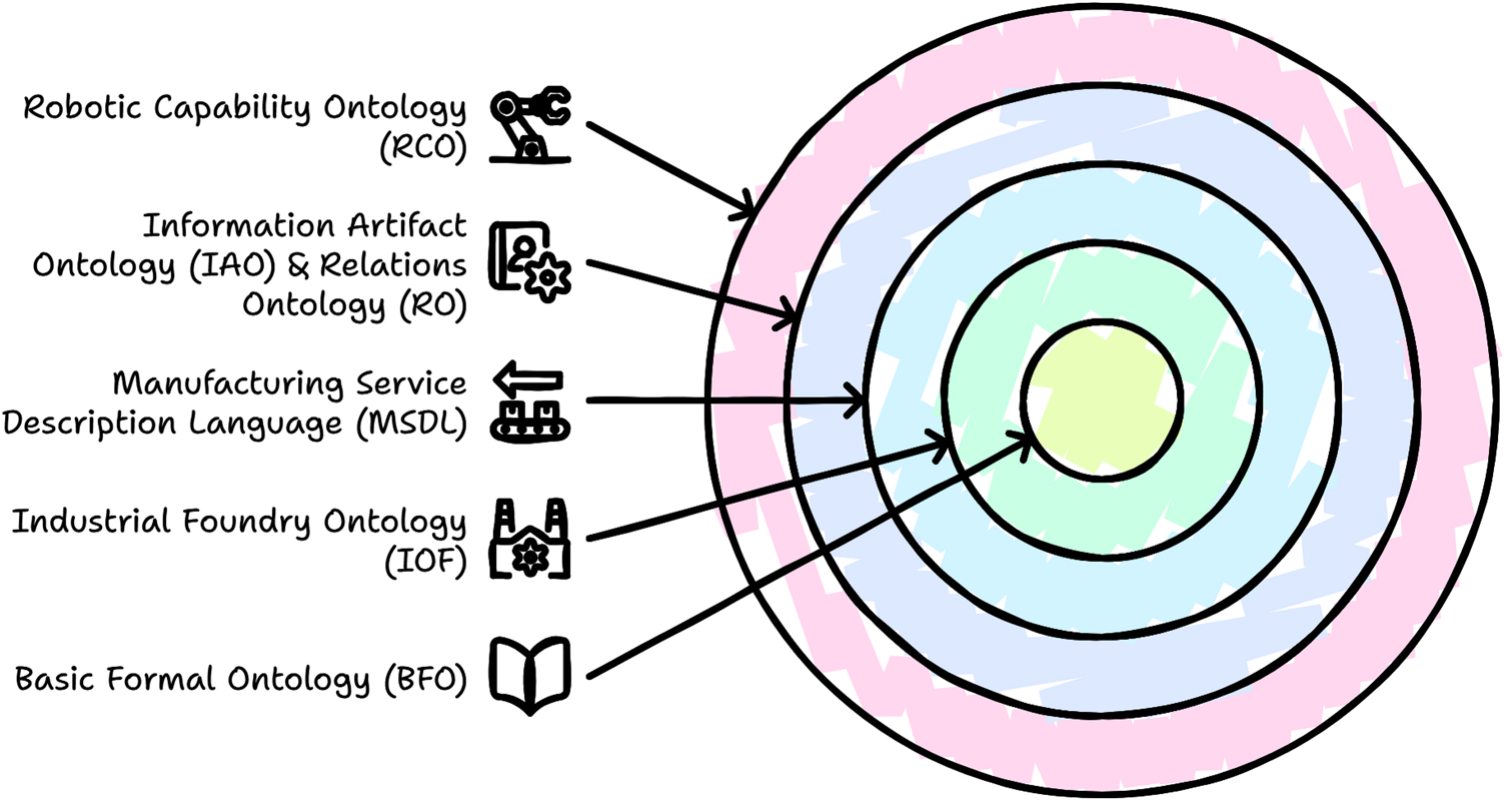
RCO hierarchical structure.

As a next step to utilize existing ontologies, RCO follows the hierarchical structure as shown in Fig. 5.

RCO based on MSDL, a domain reference ontology created for manufacturing services and aligned with the BFO, IOF, IAO, and RO. MSDL’s modular structure and domain-neutral classes allow RCO to describe and expand upon robotic capabilities accurately. Standardized terms and relationships already exist in the given in existing ontologies, giving us a strong base on which to build. Also, one of the key aspects of using “MSDL ontology is a significant model for representing manufacturing processes, as it captures more specific and rigorous manufacturing domain knowledge”^88^.

Once we identify the existing ontologies to reuse, we define the different classes and their hierarchy. Then, we define each property’s domain range, and to instantiate RCO, we utilize data from the CHAIKMAT use case. We start by importing existing concepts in RCO as we utilize different ontologies. The following section discusses the different terms we reused from existing ontologies and how we formalize our class instances and properties under the umbrella of existing ontologies.

### Modeling robot capabilities

Key concepts that we used from MSDL are the following: MSDL: Capability is “a disposition in whose realization some agent has an interest”; MSDL: Engineered Artificat refers to “an object or object aggregate that is deliberately created to have a certain function and is prescribed by some design specifications.” MSDL: Production Equipment class is a subclass of MSDL: Engineered Artifact, representing physical and digital items produced through engineering design and production techniques. We have defined the class RCO: Handling and Transportation Equipment within the MSDL:Equipment class to describe equipment specially built for material handling and transportation. we’ve introduced the class RCO: Robot as a subclass of RCO: Handling and Transportation Equipment, implying that robots are specific equipment for material handling and transportation in our scope.

As a result, instances such as the RCO:NED2 can be defined under the RCO: Robot class, indicating that it is a component of the larger domain RCO:Handling and Transportation Equipment and, subsequently, MSDL:Equipment.

**Fig. 6:**
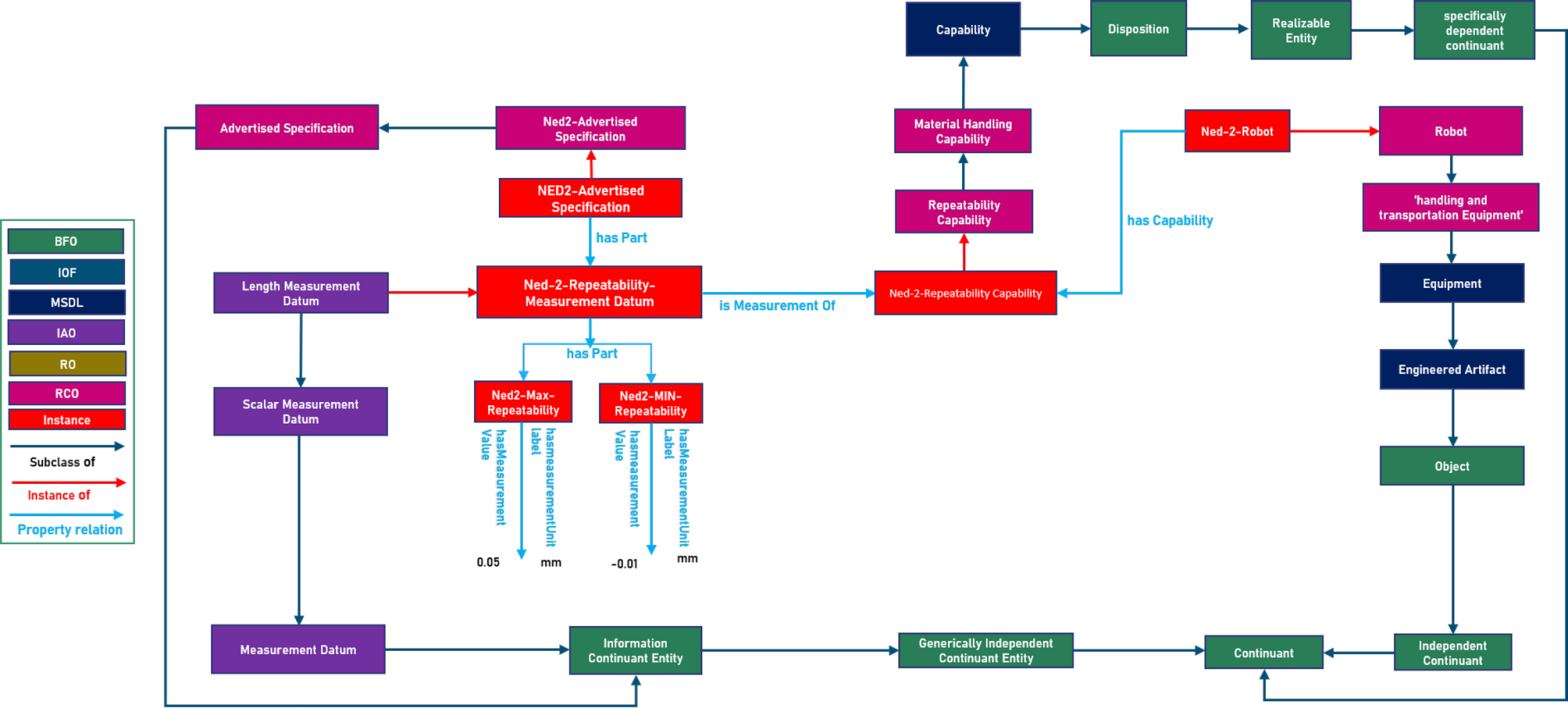
Formalization of robot capabilities.

RCO amended the MSDL’s Capability class, a subclass of the BFO:Disposition class of BFO: Entity, to depict distinct robot capabilities explicitly. This class relates to an objective, representing an entity’s ability to achieve targets connected with its overall purpose. We added the RCO:Material Handling Capability as a subclass of MSDL:Manufacturing Capability. Subsequently, the RCO: Repeatability Capability class is a subclass of Material Handling Capability as shown in Fig. 6^87^.

The IAO: Measurement Datum class is a generic class representing measurement data.IAO: Scalar Measurement Datum is a subclass of IAO: Measurement Datum, and IAO: Length Measurement Datum is a subclass of IAO: Scalar Measurement Datum to define length-related measurement data properly.

To model the repeatability measurement values, we created RCO: NED2 Advertised Repeatability Measurement and RCO: NED2 Operational Repeatability Measurement as instances of class IAO: Length Measurement Datum to capture the repeatability capability measurement value. The object attribute MSDL:has Capability, which links entities to their corresponding capabilities.

Thus, using the MSDL:hasCapability , object property, the RCO: NED2-Robot instance is connected to the RCO: Ned2 Advertised Repeatability Capability and RCO:Ned2 Operational Repeatability Capability.

Moreover, the object attribute RCO: is Measurement Of represents the link between measurements and capabilities.

IAO: Measurement Datum, a subclass of BFO: Information Continuant Entity, is the domain of the RCO:is Measurement Of, and the range this object property is ’BFO: specifically Independent Continuant, representing the measurement’s related specific independent continuant entity.

BFO relationship BFO_0000051 (has_Part) is the inverse relation of BFO:part Of represents the lowest and highest values of the NED2 repeatability capability and transmits measurement values, measurement data, and parameter units. The property IOA:has measurement Unit Label shows the parameter unit’s label, whereas IOA:has Measurement Value associates the measurement value with a specific datum.

The RCO extends MSDL’s structure as it can expand the collection of classes and relationships by introducing new domain-specific notions required for capturing robot operational and advertised capabilities and their measurement process.

**Fig. 7:**
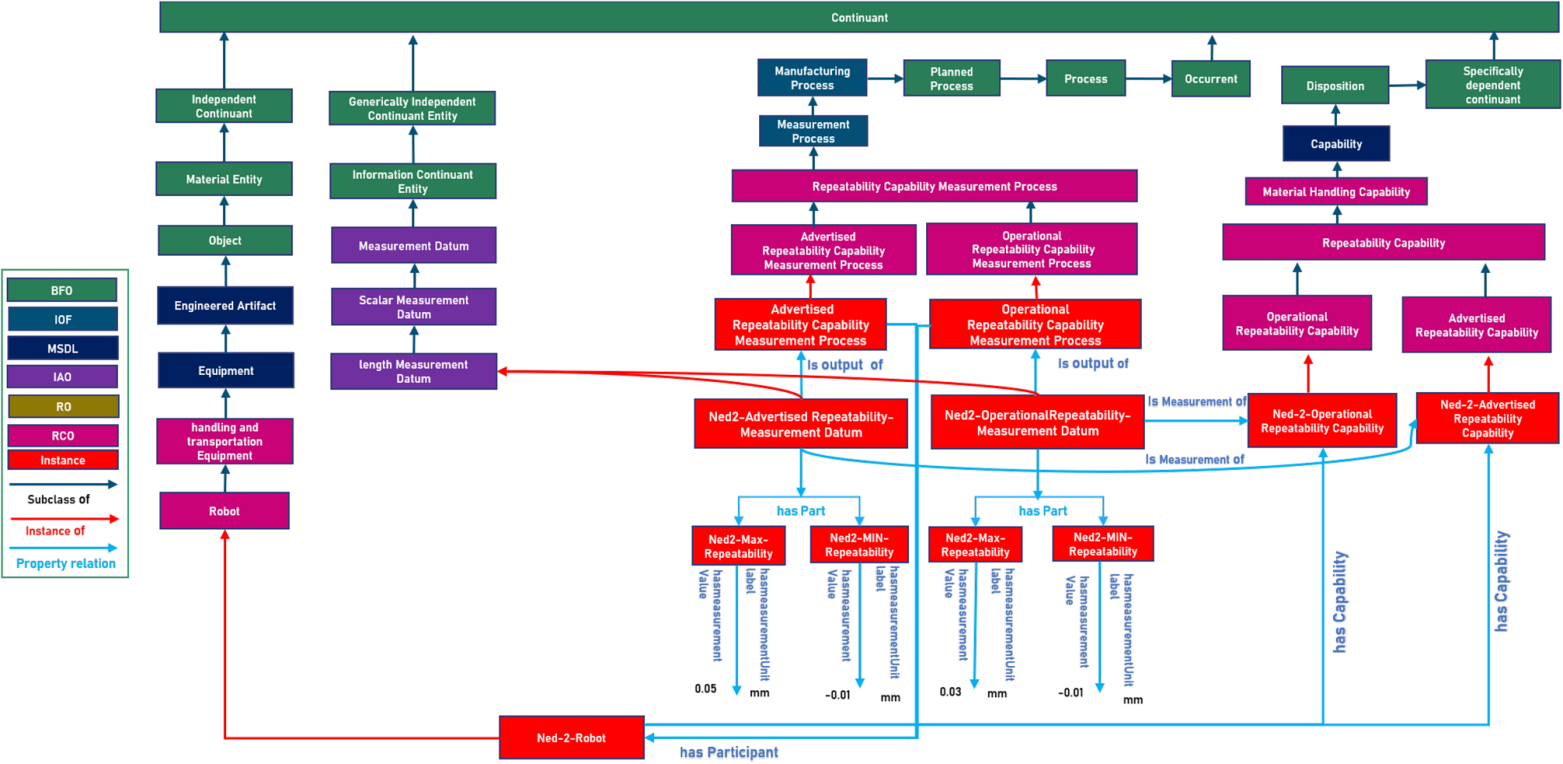
Formalization of repeatability advertised and operational capability measurement process.

We used Iof-core: measurement process, “planned process to determine the value of a quantity,” which is a subclass of Iofcore:manufacturing process “planned process that consists of a structured set of operations through which input material is transformed or modified into another material entity” we created a class RCO: Repeatability Capability Measurement Process under the class of Iof:core: Measurement Process class and then subsequently Advertised and operation capability Measurement process under the RCO: Repeatability Capability Measurement Process, as shown in Fig. 7 with example of Repeatability Capability.

#### Formalizing the notion of advertised and operational capability measurement process

To formalize the notions of advertised and operational capability measurement processes. Both advertised and operational Measurement process subclasses are formalized based on the following axioms [$\sqsubseteq$ is used to denote the subclass relationship. $\exists$ is used for existential quantification to indicate some relationship or property for the class.]:

**RCO: Advertised Capability** **Measuremen**t **Process**

- Subclass of: RCO: Capability Measurement Process
- Natural Language Definitions:
  1. Tested under controlled conditions at a manufacturer’s facility.
  2. Reflects the manufacturer’s specifications for the equipment capabilities.
- DL Axioms:
  $$\begin{aligned}&RCO:AdvertisedCapabilityMeasurementProcess \sqsubseteq RCO:CapabilityMeasurementProcess\\&\exists \text {BFO:occursIn}.ManufacturerTestingFacility \sqsubseteq RCO:AdvertisedCapabilityMeasurementProcess \\&\exists \text {BFO:environs}.ManufacturerSpecifications \sqsubseteq RCO:AdvertisedCapabilityMeasurementProcess \end{aligned}$$
- General Class Axioms:
  - RCO:AdvertisedCapabilityMeasurementProcess
    **SubClassOf** BFO:occursIn ***Some*** RCO:ManufacturerTestingFacility
    **SubClassOf** BFO:environs ***Some*** RCO:ManufacturerSpecfications

**RCO:Operational Capability Measurement Process**

- Subclass of: RCO:Capability Measurement Process
- Natural Language Definitions:
  1. Measured in an operational environment, such as a factory or real-use scenario.
  2. Reflects the equipment’s actual performance at run time in real-world conditions.
- DL Axioms:
  $$\begin{aligned}&RCO:Operational Capability Measurement Process \sqsubseteq RCO:Capability Measurement Process \\&\exists \text {BFO:occursIn}.Operational Environment \sqsubseteq RCO:Operational Capability Measurement Process \\&\exists \text {BFO:environs}.Real World Performance \sqsubseteq RCO:Operational Capability Measurement Process \end{aligned}$$
- General Class axioms:
  - RCO:OperationalCapabilityMeasurementProcess **SubClassOf** BFO:occursIn ***Some*** RCO:OperationalEnvironment **SubClassOf** BFO:environs ***Some*** RCO:operationalConditions

Manufacturer-advertised capability, referred to as RCO: Advertised Capability, is tested at a manufacturer’s facility under carefully regulated conditions and corresponds to its specifications. According to logical axioms, it is not the capability itself but the process of realizing this capability that occurs within a particular establishment BFO:occursIn and is surrounded by BFO: environs the manufacturer’s specifications or operational conditions.

RCO:Operational Capability Measurement Process class is specifically designed to evaluate the capabilities of robotic equipment in actual operational settings, such as on production lines or in real-use scenarios. It is a subclass of the broader RCO:Capability Measurement Process, emphasizing assessments carried out in the environments where the robots are intended to function, differentiating it from capabilities measured under controlled laboratory conditions.

##  Ontology validation using SPARQL

A thorough evaluation was conducted to confirm that the proposed ontology effectively serves its intended purpose in terms of coverage and completeness. Each (CQ) was validated through a corresponding SPARQL query along with their respective results.

**Figure:**
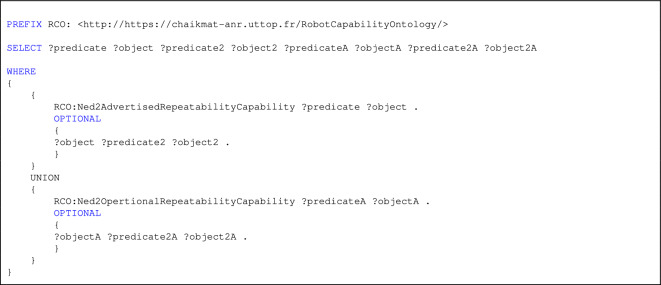
Listing 1. SPARQL Query for CQ1

CQ1, which examines the classification of advertised and operational capabilities, was validated using Query in listing 1.

Listing 1 presents a SPARQL query designed to retrieve and compare both the advertised and operational repeatability capabilities of a specific robot, modeled using the Robot Capability Ontology (RCO). The query extracts all direct properties of the advertised (RCO:Ned2AdvertisedRepeatabilityCapability) and operational (RCO:Ned2OpertionalRepeatabilityCapability) repeatability specifications, along with their related attributes.

This classification query (CQ1) is essential for validating whether a robot’s actual performance aligns with manufacturer claims. By enabling this comparison, the query supports manufacturers in identifying discrepancies between expected and real-world performance, facilitating more informed decision-making when selecting robots for precision-sensitive manufacturing tasks.

**Fig. 8:**
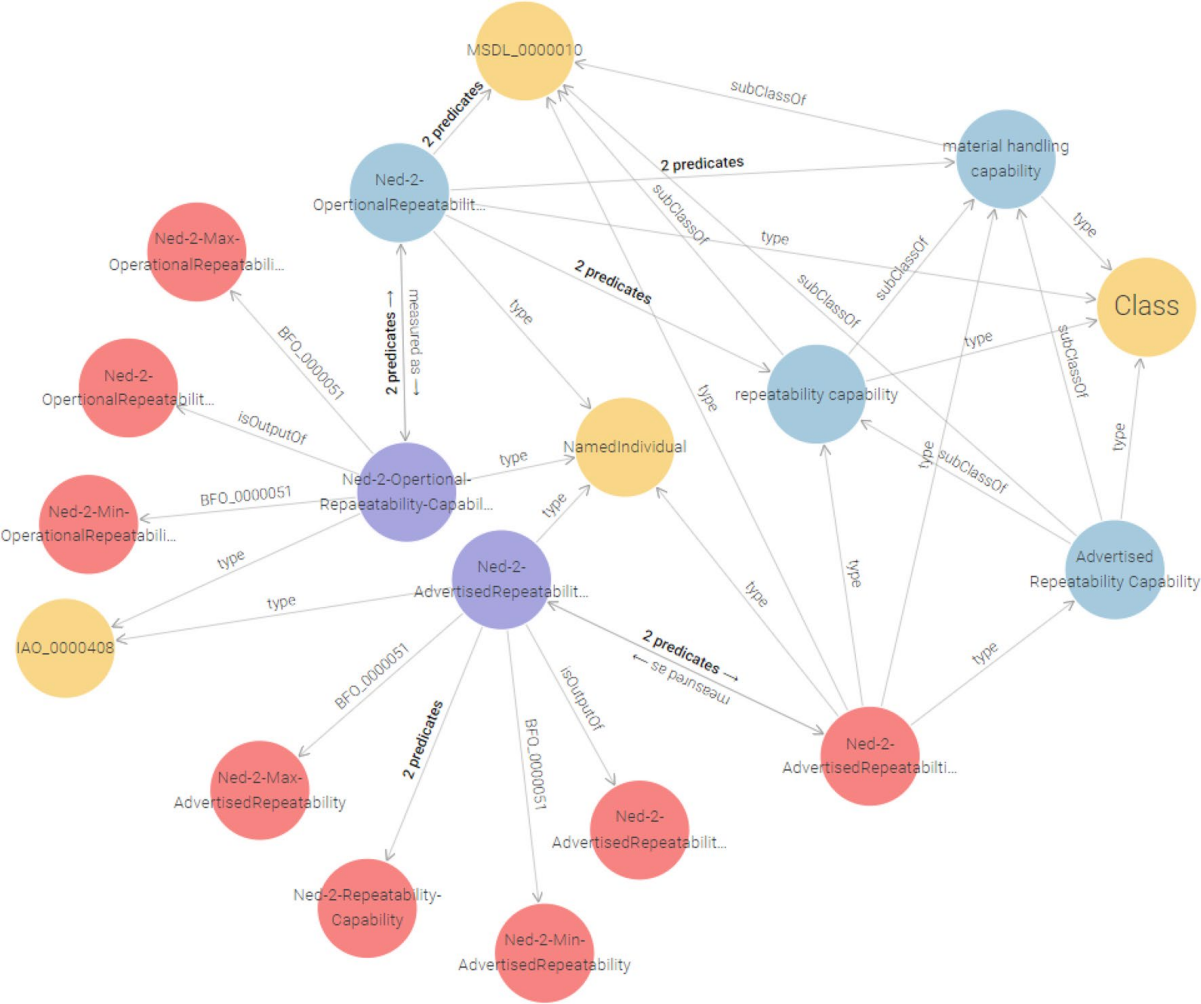
Advertised vs. operational repeatability capabilities.

Figure 8 illustrates the query results, offering a visual comparison that aids in understanding the operational reliability and suitability of robotic systems in practical settings.

Likewise, the validation of CQ2 regarding the methodology employed to assess these capabilities was validated via a specific inquiry in listing 2.

**Figure:**
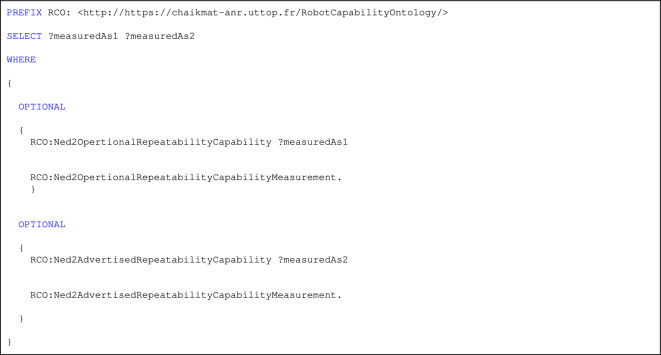
Listing 2. CQ2: Modeling advertised and operational capabilities measurement process.

The query retrieves the values linked through the measuredAs property, which quantifies repeatability in standardized units such as millimeters. This capability-to-measurement mapping is critical for manufacturers aiming to assess the accuracy and reliability of robotic systems. Specifically, it allows them to identify discrepancies between the robot’s advertised precision and its actual performance in real-world conditions. Such insights enable more informed decision-making during robot selection and deployment, particularly in manufacturing processes that demand high levels of precision.

**Fig. 9:**
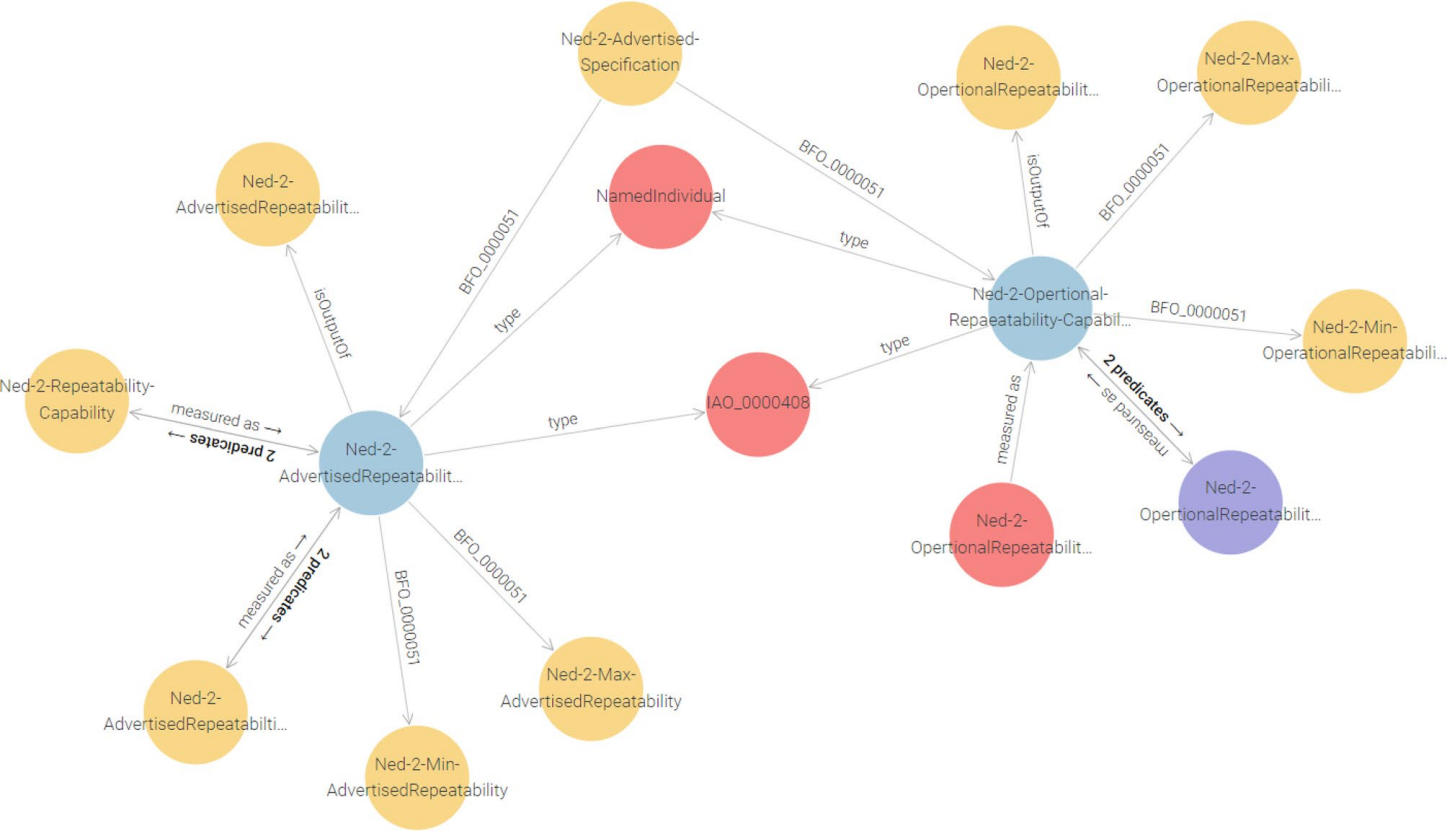
Measurement processes for advertised and operational capabilities.

It constructs relationships that describe how each type of capability, whether advertised or operational, is measured using distinct measurement nodes within the Ontology Query Results in Fig. 9.

For CQ3, The validation of information depiction methods for emphasizing differences between advertised and operational capabilities was conducted in the listing 3.

**Figure:**
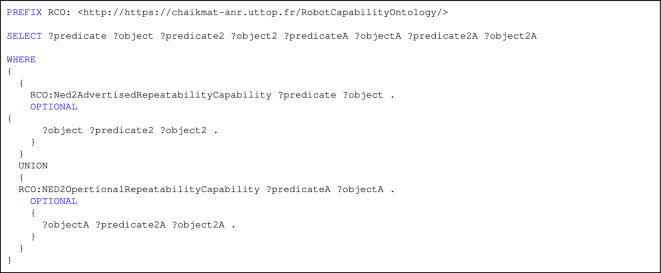
Listing 3. SPARQL query for CQ3.

**Fig. 10:**
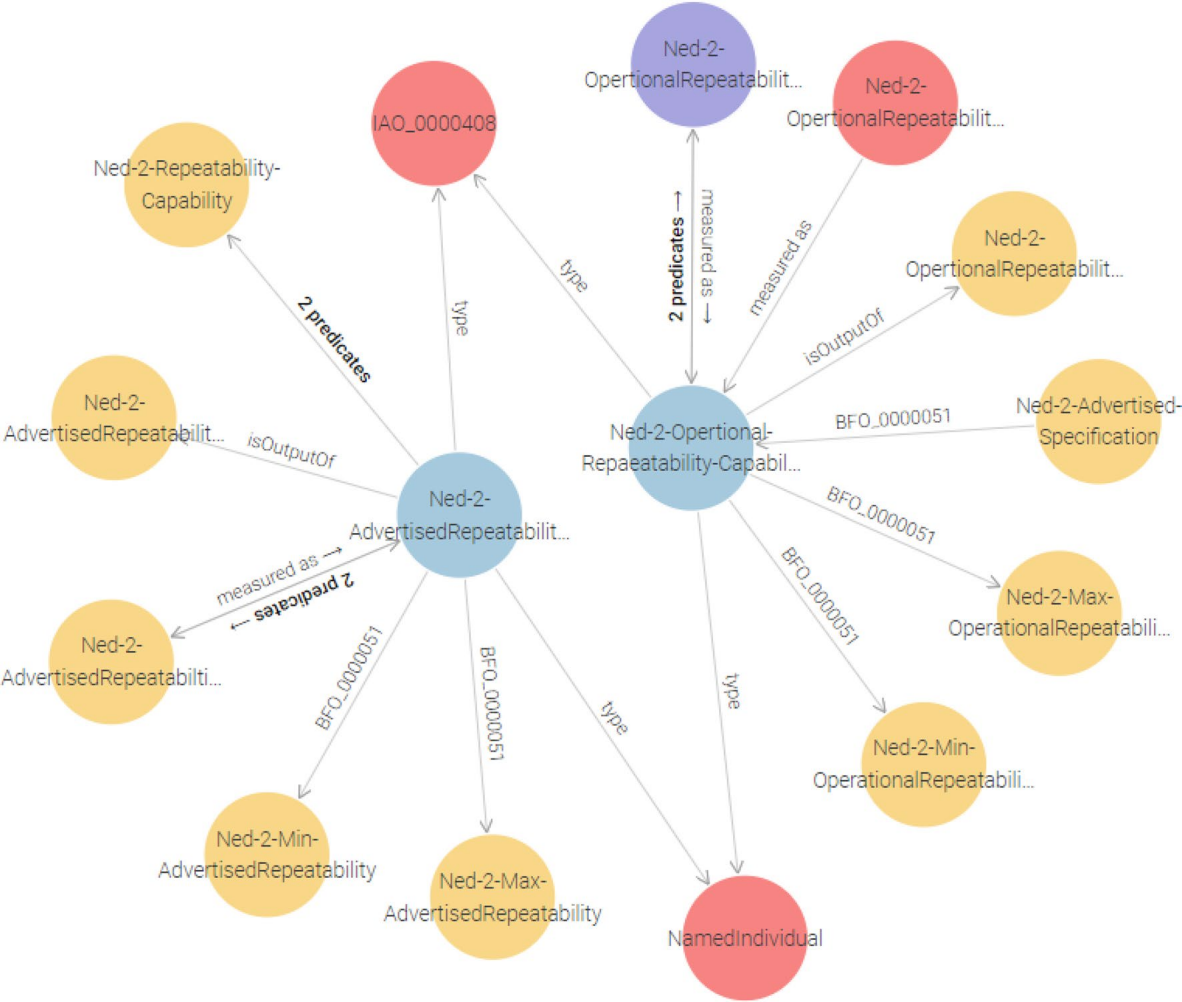
Disparities in advertised vs. operational capabilities.

Listing 3 shows a SPARQL query aimed at retrieving comprehensive descriptions of both advertised and operational repeatability capabilities for a robot. It collects all immediate properties and values related to each capability and further explores any nested relationships by including an optional second level of property-object pairs. This level of detail enables a deeper semantic comparison between what manufacturers claim (advertised capability) and what is actually observed in practice (operational capability) as shown in Fig. 10.

By enabling such structured queries over the Robot Capability Ontology (RCO), manufacturers can trace how performance claims are modeled, identify discrepancies or missing metadata, and ensure that robot capabilities are accurately understood and verifiable in context. This contributes to more transparent decision-making and enhances trust in robotic performance evaluations.

##  Application of RCO

Using the Robotic Capability Ontology (RCO) significantly enhances the formalization of robotic knowledge by providing a structured, semantically rich representation of both advertised and operational capabilities. This dual representation allows stakeholders to move beyond static vendor specifications and engage with dynamically derived, context-aware insights into actual robot performance.

Through SPARQL-based queries over the RCO model, users can efficiently retrieve and analyze capability data, as demonstrated in the NED2 robot use case. This not only reduces the manual effort involved in evaluating robots but also enables traceable, reproducible, and data-driven comparisons. The implications of this are particularly important in industrial settings, where decision-makers must assess whether a robot’s claimed specifications (e.g., repeatability, payload, precision) truly hold under specific environmental or task conditions.

By capturing both the advertised and operational performance characteristics, the RCO enables predictive assessments of performance deviations and supports the identification of systematic limitations. This mitigates risks associated with mismatched expectations and reduces failures during deployment. For example, a manufacturer can use RCO to compare task requirements-such as load variability or environmental constraints-against historical operational data, helping to anticipate under performance and guide corrective actions like task reallocation or robot substitution.

Additionally, the RCO supports continuous performance monitoring and knowledge refinement by integrating runtime observations into the knowledge base. This allows for iterative updates of robot profiles based on real-world feedback, supporting long-term optimization of robotic assets.

RCO also facilitates cross-platform comparisons by providing a standardized vocabulary and reasoning logic that enables fair benchmarking of robots from different vendors or configurations. This helps stakeholders make procurement decisions based on verifiable, ontology-aligned metrics rather than marketing claims alone.

Moreover, system integrators can leverage RCO to simulate capability matches in task planning tools, reducing integration costs and deployment delays. By modeling capability dependencies and constraints explicitly, RCO enhances early-stage validation and compatibility assessment in heterogeneous robotic systems.

Finally, RCO provides a formal basis for auditing and certification processes. Regulatory bodies and safety engineers can use the ontology to verify compliance with operational thresholds and to trace capability degradation over time, enabling robust lifecycle assessments.

Therefore, the RCO does not only serve as a knowledge formalization tool but also provides a reasoning framework that directly contributes to narrowing the gap between advertised capabilities and real-world performance. It enables more reliable robot-task matching, supports transparency in procurement, and offers a foundation for building adaptive systems that account for operational uncertainty.

## Conclusion

Our work demonstrates the significance of ontological frameworks in accurately capturing robotic capabilities to support industrial flexibility and to differentiate between advertised and operational capabilities, alongside function quality and process characteristics of robots. The study underlines the importance of semantically precise frameworks, as robots can perform a broader range of functions. An ontological framework enables a thorough comprehension of concepts like reach, accuracy, repetition, and various robotic behaviors. These are critical in selecting the appropriate robots for specific jobs, enhancing productivity, reducing inefficient usage and potential dangers, and promoting trustworthy and adaptable production.

Overall, using an ontological framework in robotic capability advances flexible production systems and improves the creation and use of autonomous robots. Our future work will focus on formalizing notions such as the different roles of robots in task planning and the function, quality, and role of robots to enhance understanding and utilization of robotic capabilities in flexible manufacturing systems, including knowledge-driven explanations of where robots are specifically being deployed.

Our future work will focus on integrating the Robotic Capability Ontology (RCO) with real-time predictions from a neural network to support robot selection based on operational capabilities. The neural network will predict performance characteristics at runtime, while the RCO will be used to reason over these predictions using structured queries. This combination will allow the system to identify suitable, unsuitable, and optimal robots for a given task, enabling more informed and flexible decision-making in dynamic manufacturing environments.

## Data Availability

The datasets generated and/or analysed during the current study are not publicly available but are available from the corresponding author on reasonable request.
